# Low relative muscle volume: Correlation with prevalence of venous thromboembolism following total knee arthroplasty

**DOI:** 10.1371/journal.pone.0210800

**Published:** 2019-03-05

**Authors:** Jung-Min Shin, Su-Jin Hong, Kyung-Hwa Choi, Sung-Il Shin, Do Kyung Lee, Sung-Sahn Lee, Byung Hoon Lee

**Affiliations:** 1 Department of Internal Medicine, Hanyang Medical Center, Hanyang University College of Medicine, Seoul, Republic of Korea; 2 Department of Radiology, Hanyang University Guri Hospital, Gyeonggi-do, Republic of Korea; 3 Department of Preventive Medicine, Dankook University College of Medicine, Cheonan-si, South Korea; 4 Department of Orthopaedic Surgery, Kang-Dong Sacred Heart Hospital, Hallym University Medical Center, Seoul, South Korea; 5 Department of Orthopedic Surgery, Konyang University Hospital, Konyang University School of Medicine, Daejeon-si, Chungcheongnam-do, Republic of Korea; 6 Department of Orthopedic Surgery, Samsung Medical Center, Sungkyunkwan University School of Medicine, Seoul, Republic of Korea; Medical University Innsbruck, AUSTRIA

## Abstract

**Background:**

There have been many efforts to find modifiable risk factors for venous thromboembolism (VTE) in the perioperative period of total knee arthroplasty (TKA), while no study has investigated the relationship between the muscle mass and deep vein thrombosis (DVT) or pulmonary embolism frequency following TKA. This study aimed to evaluate the relationship between muscle volume and the prevalence of symptomatic and radiologically confirmed venous thromboembolism (VTE) after total knee arthroplasty (TKA).

**Methods:**

A total of 261 consecutive patients who underwent primary TKA between 2013 and 2015 were enrolled. Computed tomographic venography with pulmonary angiography (CTVPA) was performed between the 5th and 7th postoperative days to assess the presence of VTE. Four parameters of muscle volume at three levels were evaluated on CTVPA: (i) the cross-sectional area of all skeletal muscles (skeletal muscle index) and total psoas area at the level of the third lumbar vertebrae; (ii) the vastus lateralis muscle at the thigh level; and (iii) the posterior crural muscle at the lower leg level. The relationship between the muscle volume at each level and the prevalence of VTE after TKA was evaluated with multivariate adjusted logistic regression models.

**Results:**

The CTVPA scan showed no proximal DVT, and all thrombi were located in muscular, peroneal, and posterior tibial veins. In unilateral TKA, patients with lower muscle volume of the vastus lateralis at the thigh level in the nonoperated limb had significantly higher prevalence of distal DVT in the operated limb (adjusted OR: 2.97 at subclinical DVT revealed by CTVPA and adjusted OR: 2.68 at symptomatic DVT). This finding was also discovered in patients who underwent simultaneous bilateral TKA (adjusted OR: 1.73–2.97 at subclinical DVT and adjusted OR:1.76–1.86 at symptomatic DVT).

**Conclusions:**

The relative muscle volume of the vastus lateralis at the thigh level was negatively associated with the prevalence of symptomatic and radiologically confirmed DVT, suggesting that low thigh muscle mass is an independent risk factor for VTE in the postoperative period of TKA.

## Introduction

Venous thromboembolism (VTE), which includes deep vein thrombosis (DVT) and pulmonary embolism (PE), is a major cause of morbidity and mortality in postoperative hospitalized patients. DVT can frequently lead to PE, a fatal complication after arthroplasty [1,2] As described in detail previously [3], the frequency of VTE can increase after total knee arthroplasty (TKA) owing to increased blood clotting tendency, which results from low blood flow, ischemic tissue damage, and blood vessel damage due to intraoperative hemostasis with an overbent knee position and tourniquet use.

To improve the vascular flow and decrease venous stasis in the soleal vein during surgery, and to reduce the DVT incidence after TKA, mechanical measures (early ambulation and active mobilization, use of thigh-high antiembolic stockings, intermittent pneumatic compression, and mechanical calf muscle stimulation) are attractive options [4]. To maximize the mechanical measures for allowing early mobilization, such as quadriceps strengthening and active ankle pump exercises for DVT prophylaxis, large muscle volume and increased performance would be advantageous. However, muscle atrophy and declining strength inevitably occur with aging. Other cross-sectional studies reported that muscle performance reduction is a direct result of decreased muscle mass. Indices of frailty, such as sarcopenia, can be diagnosed through the measurement of the physical volume of muscle [5]. Low muscle mass has negative correlations with an increased prevalence of coronary arterial disorder and metabolic syndrome [6].

Nevertheless, to our knowledge, no study has investigated the relationship between muscle mass and the frequency of DVT or PE after TKA. We hypothesized that muscle mass might be a modifiable perioperative predisposing factor for DVT. This study was conducted to identify the prevalence of VTE, especially symptomatic and radiologically confirmed DVT and PE, among patients after TKA by using computed tomographic venography with pulmonary angiography (CTVPA) performed between the 5th and 7th postoperative days, which is considered the gold standard for the precise detection of thromboembolic lesions. Therefore, the purpose of this study was to evaluate the relationship between muscle volume and the prevalence of symptomatic and radiologically confirmed DVT or PE after TKA.

## Materials and methods

The present study was approved by the institutional review board, and signed informed consent was obtained from each participant (Ethics Committee Boards of Kangdong Sacred Hospital, ID number: KDF 2016-10-011). We enrolled 315 consecutive patients admitted to our institution for primary TKA between March 2013 and March 2015. Patients diagnosed as having osteoarthritis were included. If a patient received staged bilateral TKA during this period, only the first procedure was included. Only patients with complete follow-up data for a minimum of 2 years were selected.

**Fig 1:**
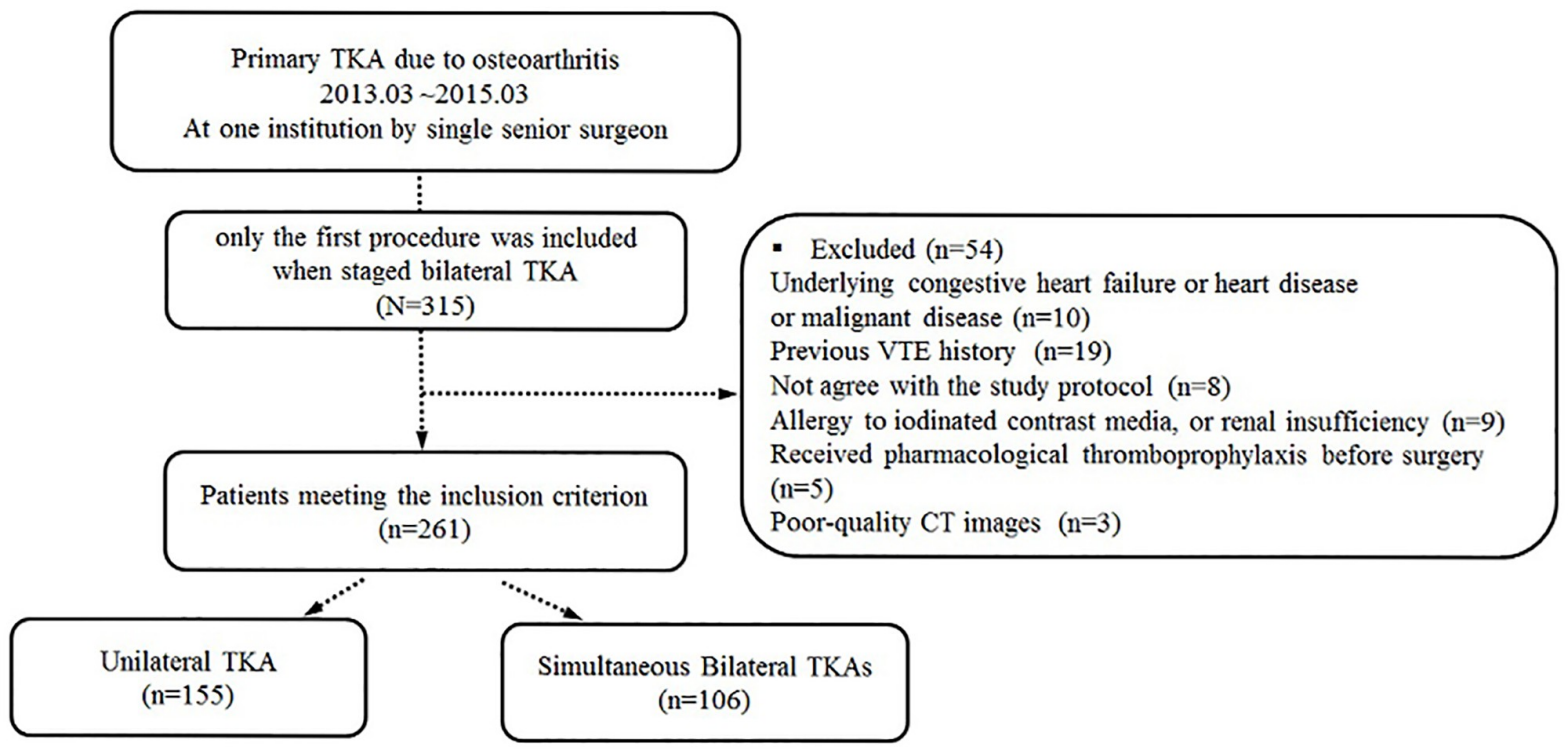
Patient flowchart. CT, computed tomography; DVT, deep vein thrombosis; TKA, total knee arthroplasty VTE, venous thromboembolism.

The following patients were excluded: (i) with previous venous disease (including VTE history), congestive heart failure or heart disease or both, malignant disease, previous or current estrogen therapy, or genetic and dietary factors that increase the risk of DVT [7]; (ii) with an allergy to iodinated contrast media, or renal insufficiency; (iii) who received pharmacological thromboprophylaxis before surgery; and (iv) who did not agree with the study protocol or with poor-quality computed tomography (CT) images (Fig 1).

All patients aged >60 years underwent 2D echocardiography to confirm their cardiovascular status. Patients were assessed by an anesthesiologist, and their disease was classified according to the American Society of Anesthesiologists (ASA) grading system. If any significant comorbidity was identified, the patient was referred to a specialist for the assessment of fitness for surgery. No patient with previous adverse cardiopulmonary events (myocardial infarction, PE history) or ASA grade 3 or 4 was offered simultaneous bilateral surgery [3].

All surgeries were performed by single senior surgeon (one of the authors). As described in detail previously [8], allogeneic blood transfusion was performed if the hemoglobin level decreased to <8.0 g/dL or if anemic symptoms, such as dyspnea or tachycardia, persisted even after volume replacement in patients with a hemoglobin level between 7.0 and 8.0 g/dL [9]. All patients received pneumatic compression system twice a day for 30 min and were instructed to perform repeated dorsiflexion and plantar flexion of the ankles while awake, as soon as they were returned to the ward. Quadriceps-strengthening exercises were started immediately after surgery as part of a basic postoperative rehabilitation program, and continuous passive motion exercises were started on the first postoperative day. Ambulation was allowed on the second postoperative day, after drainage removal. Thereafter, active and passive joint exercises were allowed within a comfortable range of motion.

All clinical and radiographic evaluations were performed by an independent investigator, and perioperative in-hospital complications were recorded at each follow-up visit scheduled at 2, 6 months, 1 year, and annually thereafter. All participants received the recommended thromboprophylaxis regimen (with a low-molecular-weight heparin) from the operative day to the day of CTVPA. CTVPA was performed in all patients to detect the presence of DVT or PE during 5–7 days after the operation. Any symptoms suggestive of DVT, such as calf pain, increased calf circumference, and presence of Homan’s sign, were evaluated daily in the postoperative period until discharge. Any shortness of breath, chest pain, or blood-streaked sputum suggestive of PE was also evaluated. Patients were instructed about the symptoms of DVT or PE, and were advised to contact the hospital in case any of these symptoms develop after their discharge. All patients were followed for 2 years after the surgery.

**Fig 2:**
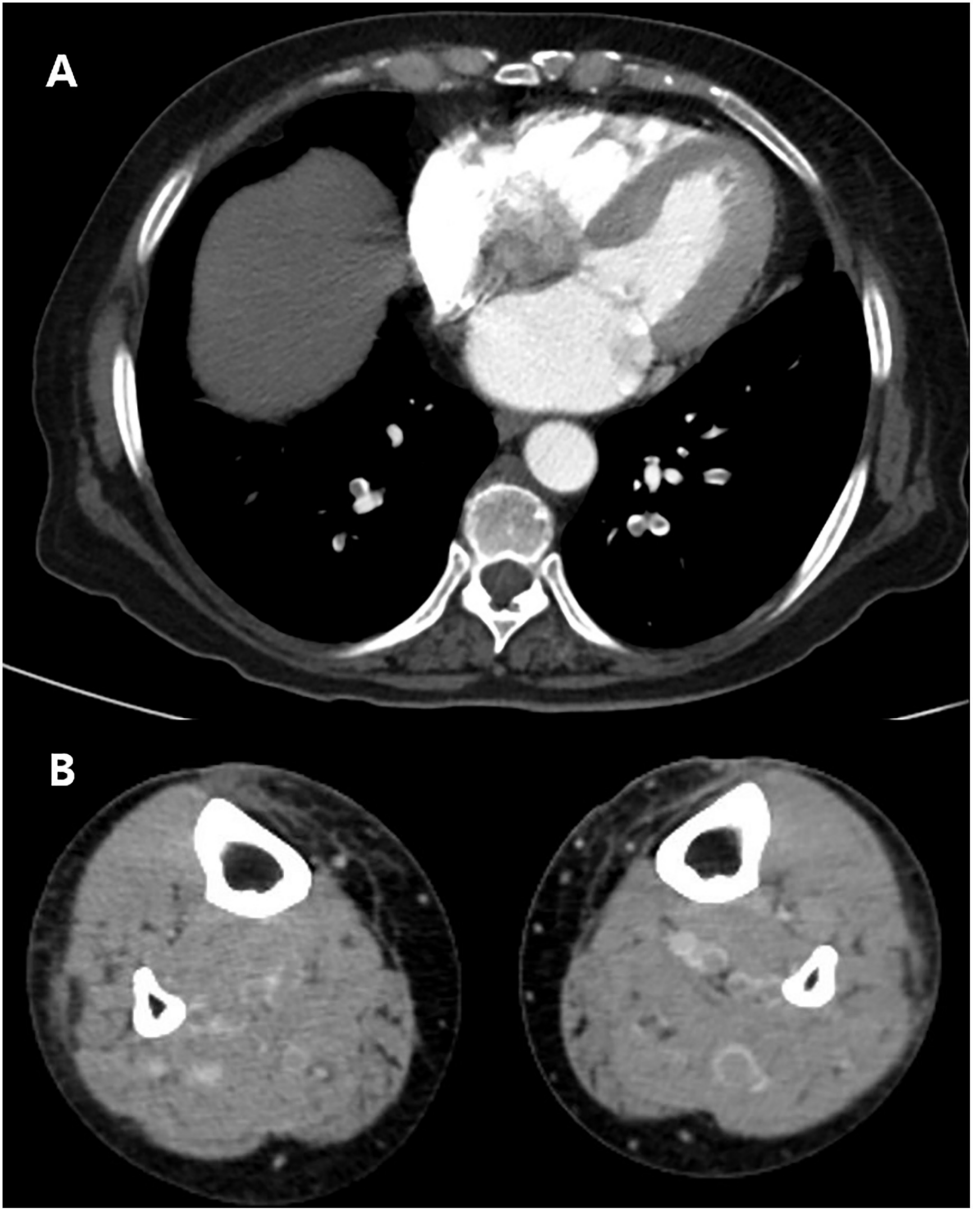
(a) Pulmonary embolism in both subsegmental pulmonary arteries of both lower lobes (b) Deep vein thrombosis in both calf veins.

The diagnosis of DVT or PE was made by a radiologist on the basis of the CTVPA findings. CTVPA scans were performed by using 256 multidetector CT scanners (iCT; Philips Medical Systems, Best, The Netherlands). On the CT scan, PE was diagnosed when a sharply delineated pulmonary arterial filling defect, located centrally within the vessel or with acute angle to the vessel wall, was observed in at least two consecutive transverse images (Fig 2A) [10]. DVT was defined as a low-attenuating partial or complete intraluminal filling defect surrounded by a high-attenuating ring of enhanced blood found on at least two consecutive transverse images (Fig 2B) [11]. The results of these imaging tests and the symptoms related to DVT or PE during hospitalization were recorded.

### Assessment of muscle volume from CTVPA

**Fig 3:**
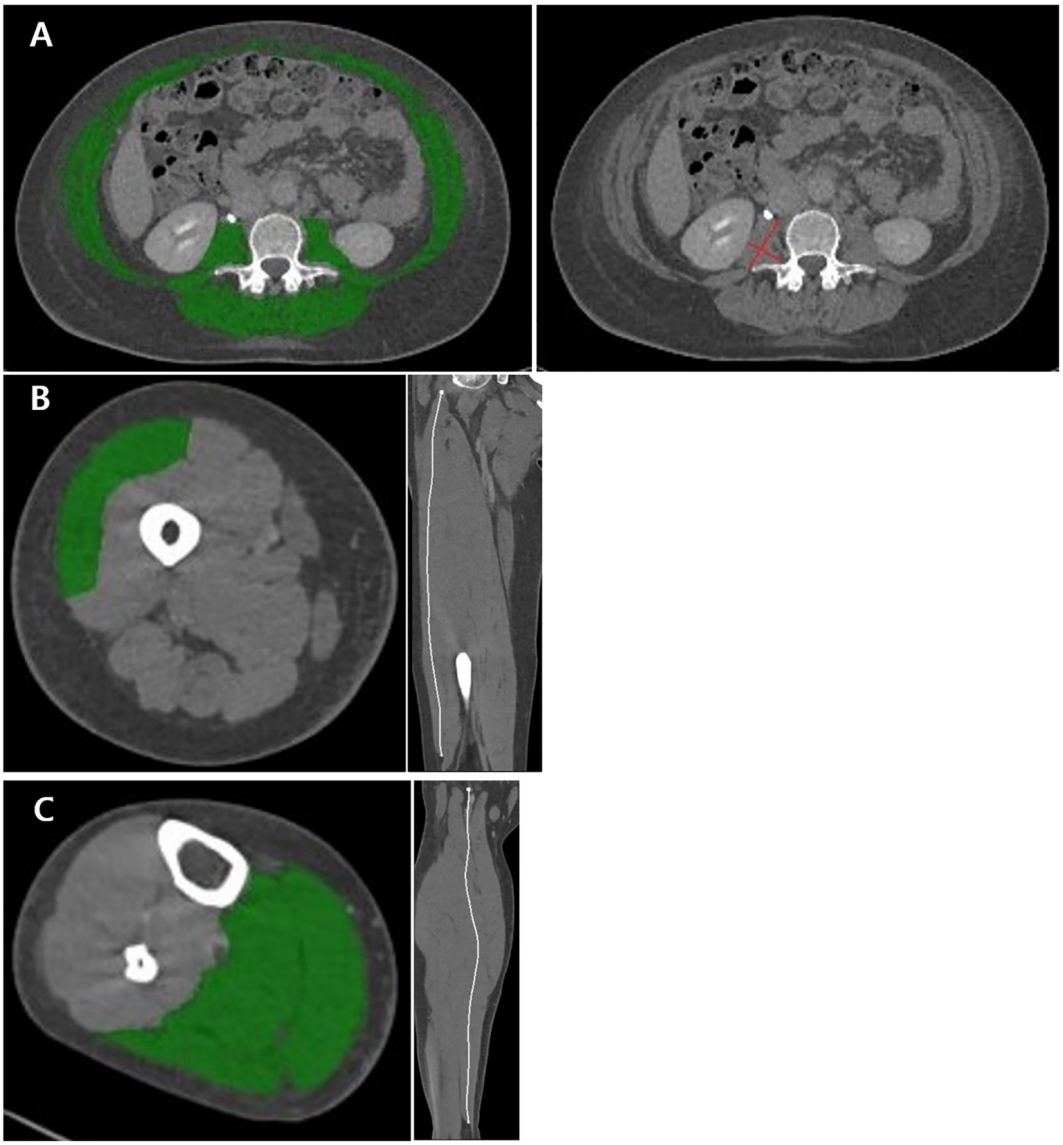
(a) Skeletal muscle index (left) and total psoas area (right) at the L3 level (b) Cross-sectional area (left) and total muscle length (right) of the right vastus lateralis muscle (c) Cross-sectional area (left) and total muscle length (right) of the right posterior crural muscles (i.e., soleus, SO; gastrocnemius medialis, GM; gastrocnemius lateralis, GL) at the calf level.

A radiologist who was blinded to patient outcomes performed quantitative assessments of the following four parameters by using commercially available software (Tera Recon, San Mateo, CA, USA): skeletal muscle index (SMI), total psoas area (TPA), volume of the vastus lateralis muscle at the thigh level, and volume of the posterior crural muscle (included with triceps surae) at the lower leg level, which was described by Taguchi et al [12]. For SMI and TPA, a single axial image that included both transverse processes at the level of the third lumbar vertebra was selected. SMI was automatically calculated by summing the appropriate pixels with the CT Hounsfield unit range -29 to 150 HU for all skeletal muscles [13]. Psoas muscle thickness was manually assessed by measuring the largest diameter of the muscle for axial thickness and the diameter of muscle perpendicular to the axial diameter for transverse thickness. Subsequently, TPA was calculated by multiplying the axial and transverse thicknesses [14]. The obtained values were normalized according to height squared and reported as cm^2^/m^2^ for SMI and as mm^2^/m^2^ for TPA. Fig 3A shows how these two parameters were measured on representative axial CT images at the L3 levels of the same patient. The volume of the vastus lateralis muscle at the thigh level (Fig 3B) and the volume of the triceps surae muscle (i.e., soleus, gastrocnemius medialis, and gastrocnemius lateralis) at the calf level (Fig 3C) were calculated by using a simplified assessment method with the muscle length and its maximum cross-sectional area (CSA) [15].

Total muscle length was measured by using a curved multiplanar reconstruction view, which was generated by connecting the origin and insertion of each muscle. The boundaries of the muscles were tracked manually, and CSAs were automatically measured after removing the “marbling part” in the same way as in SMI measurement. Thus, muscle volume was calculated by multiplying the maximum CSA for the muscle with the length of the muscle [15].

### Statistical analysis

The difference of distribution of the surgical side according to general characteristics was assessed with chi-square test or Fisher’s exact test. The difference of the mean of muscle volume according to general characteristics was assessed by using analysis of variance or t-test. Odds ratios (ORs) and 95% confidence intervals (CIs) were estimated by using logistic regression models adjusted for confounding factors. Confounding factors, such as age, sex, and BMI were considered significant factors in the analyzed difference of the distribution of muscle volume and DVT. We categorized patients according to age (<60, 60–69, 70–79, or ≥80 years) and BMI (<25, 25–30, or ≥30 kg/m^2^). All statistical analyses were performed with R 3.1.2 (available from http://www.r-project.org/). The significance level was set at 0.05.

## Results

A total of 261 patients were enrolled in the study after excluding 27 patients who refused VTE screening or with a known VTE history, 10 patients with predisposing factors to DVT due to previous venous disease, 9 patients with an allergy to iodinated contrast media or renal insufficiency, 5 patients taking anticoagulants owing to previous brain infarcts or coronary heart disease, and 3 patients with poor-quality CTVPA. Unilateral TKA was performed in 155 patients, and bilateral TKA was performed in 106 patients. The baseline characteristics of the patients are summarized in Table 1.

**Table 1:** Distribution of surgical side and muscle volume according to general characteristics (N = 261)^a^.

	All	Surgical side, n (%)			SMI (mm^2^)	TPA (mm^2^)	V lateralis m vol (mm^3^)		Post. crural m vol (mm^3^)	
		Right	Left	Both			Right	Left	Rt	Lt
All	261	71 (27.1)	84 (32.1)	106 (40.5)	607.1 (274.1)	367.4 (116.2)	38.7 (9.7)	51.8 (10.2)	1681.7 (738.5)	1800.8 (764.3)
Sex										
Male	32	10 (31.3)	16 (50)	6 (18.8)	622.8 (162)	445 (97.2)	43.8 (8.9)	64.4 (10.7)	2063.8 (1351.9)	2436.9 (1273)
Female	229	62 (27.1)	68 (29.7)	99 (43.2)	604.9 (286.7)	356.6 (114.9)	38 (9.6)	50 (8.9)	1628.5 (598.5)	1712.3 (623.1)
p-Value				0.14	0.72	**0.003**	**0.02**	**<0.0001**	**0.022**	**0.04**
Age group (years)										
<60	26	8 (30.8)	12 (46.2)	6 (23.1)	849.5 (724.9)	402.7 (145)	55 (8.7)	55.5 (13.1)	2161 (1404.1)	2237.8 (1394.1)
60–69	94	28 (29.8)	40 (42.6)	26 (27.7)	582.3 (174.5)	377.9 (115.4)	48.6 (9.3)	53.2 (11.3)	1716.7 (750.6)	1900 (799.6)
70–79	100	26 (26)	22 (22)	52 (52)	579.5 (131.9)	362.3 (107.5)	46.7 (8.8)	50.5 (8.4)	1537.6 (507.2)	1633.1 (537.9)
≥80	41	10 (24.4)	10 (24.4)	21 (51.3)	578.3 (134.5)	333.8 (117.7)	45.6 (11.8)	49.3 (9.5)	1649.8 (480.9)	1707.4 (474.3)
p-Value				0.12	**0.04**	0.07	**0.04**	**0.03**	**0.04**	**0.02**
BMI group (kg/m^2^)										
<25	96	24 (25)	35 (36.5)	37 (38.5)	533.4 (145.5)	345.9 (119.5)	36.7 (8.6)	50 (10.2)	1542.6 (846.5)	1661.3 (880)
25–<30	132	46 (34.8)	40 (30.3)	46 (34.8)	626 (145.5)	376.4 (111.4)	39.5 (10.3)	52.3 (10.7)	1699.4 (663.1)	1854.8 (700)
30+	33	2 (6.1)	10 (30.3)	21 (63.6)	741.8 (652.2)	392.7 (122.2)	41.3 (9.5)	54.8 (7.7)	2006 (607.8)	1984.9 (611.9)
p-Value				0.10	**0.005**	0.10	0.06	0.08	**0.03**	0.09
Anesthesia type										
General	190	42 (22.1)	54 (28.4)	94 (49.5)	609.5 (308.4)	370.1 (120.2)	38.8 (9.4)	52.1 (10.2)	1706.1 (833.3)	1805.6 (852.3)
Regional	71	30 (42.3)	30 (42.3)	11 (15.5)	600.7 (153.9)	360.2 (106.3)	38.5 (10.6)	51.1 (10.3)	1617.4 (393.6)	1788.1 (467.7)
p-Value				**0.001**	0.83	0.65	0.90	0.63	0.41	0.88
ASA classification										
1	30	16 (53.3)	6 (20)	8 (26.7)	769.3 (692.9)	344.3 (116.5)	40.2 (9.4)	50.5 (9.3)	1561.7 (406)	1559.7 (435.2)
2	225	55 (24.4)	76 (33.8)	94 (41.8)	582.9 (148.5)	368 (116.2)	38.2 (9.7)	51.9 (10.5)	1700.5 (780.3)	1831.8 (801.2)
3	6	0 (0)	2 (33.3)	4 (66.7)	706.8 (209.7)	456.7 (105.6)	49.1 (2.9)	53.2 (3.4)	1572.4 (232.1)	1838.6 (452)
p-Value				0.15	0.06	0.20	0.83	0.57	0.63	0.24
Comorbidity										
HTN										
No	90	34 (37.8)	30 (33.3)	26 (28.9)	652.1 (421.3)	356.3 (112.9)	39.1 (10.4)	52.4 (9.6)	1559.7 (516.9)	1649.4 (512.2)
Yes	171	38 (22.2)	54 (31.6)	79 (46.2)	583.6 (146.3)	373.1 (118.1)	38.5 (9.4)	51.5 (10.6)	1745.5 (827)	1880 (859.7)
p-Value				0.09	0.30	0.43	0.73	0.61	0.12	0.05
DM										
No	191	58 (30.2)	58 (30.2)	75 (39.3)	620 (305.1)	376.9 (115.7)	38.7 (9.8)	52.6 (10.2)	1719.4 (794.4)	1821.1 (836.3)
Yes	70	14 (20)	26 (37.1)	30 (42.9)	571.8 (159.5)	341.2 (115.1)	38.7 (9.5)	49.6 (10.1)	1578.2 (554.3)	1744.9 (524.5)
p-Value				0.48	0.25	0.12	0.998	0.14	0.26	0.54
Transfusion amount^a^	2.71 (2.13)									
0–<1	74	30 (40.5)	32 (43.2)	12 (16.2)	613.2 (164.1)	356.5 (123.8)	41.9 (9.9)	50.5 (10.8)	1644.5 (805.5)	1801 (827.8)
1–<2	78	36 (46.2)	28 (35.9)	14 (17.9)	573.9 (156.1)	349.7 (122.7)	38.1 (10.9)	51.1 (10.1)	1720.1 (499.2)	1710.2 (534.4)
2–<4	56	4 (7.1)	16 (28.6)	36 (64.3)	668.5 (516.1)	394.1 (99)	37 (7.8)	55.3 (9.2)	1843.1 (1096.8)	1959.3 (1075.5)
4–9	53	2 (3.7)	8 (14.8)	43 (81.1)	583.2 (141.1)	380 (111.9)	36.9 (8.6)	51 (10.3)	1509.9 (390.2)	1767 (559.5)
p-Value				**<0.001**	0.54	0.39	0.12	0.24	0.40	0.62

^a^ Mean (standard deviation).

p-Values <0.05 are displayed in bold.

SMI, skeletal muscle index; TPA, total psoas area; V lateralis m vol, vastus lateralis muscle volume; Post. crural m vol, posterior crural muscle volume; BMI, body mass index; ASA, American Society of Anesthesiologists; HTN, hypertension; DM, diabetes mellitus.

The TPA, volume of the vastus lateralis, and volume of the posterior crural muscles were significantly higher in men. The SMI, volume of the vastus lateralis, and volume of the posterior crural muscle decreased with age, suggesting a sarcopenic condition (age-associated loss of skeletal muscle mass [5]). Although no statistically significant difference was found among all four parameters, an increasing tendency of muscle mass was associated with increasing BMI. No significant relationship was found between each muscle volume and ASA grading (Table 1).

The incidences of symptomatic DVT and PE after TKA were approximately 7.7% and 1.1%, and the prevalence of radiologically proven DVT and PE identified from CTVPA was 11.9% (31 of 261) and 13.4% (35 of 261), respectively. The probability of symptomatic DVT and PE was higher after simultaneous bilateral TKA (adjusted OR: 0.43–0.74); however, this finding was not statistically significant.

The CTVPA scan showed no proximal DVT, and all thrombi were located in muscular, peroneal, and posterior tibial veins. The incidence of radiologically confirmed DVT was significantly lower in unilateral TKA than in simultaneous bilateral TKA (adjusted OR: 0.20 in the right side, 0.72 in the left side).

In unilateral TKA, patients with lower muscle volume of the vastus lateralis at the thigh level in the nonoperated limb had significantly higher prevalence of distal DVT in the operated limb (adjusted OR: 2.97 at subclinical DVT revealed by CTVPA and adjusted OR: 2.68 at symptomatic DVT). This finding was also discovered in patients who underwent simultaneous bilateral TKA (adjusted OR: 1.73–2.97 at subclinical DVT and adjusted OR:1.76–1.86 at symptomatic DVT). However, we could not confirm whether lower SMI or TPA significantly increased the risk of PE (Tables 2 and 3) (More details were reported in S1 Table).

**Table 2:** aORs and 95% CIs of radiologically confirmed and symptomatic VTE according to muscle volume in unilateral TKA.

		Radiologically confirmed PE		Radiologically confirmed DVT at operated limb		Radiologically confirmed DVT at unoperated limb		Symptomatic DVT	
	n(155)	n(19)	aOR (95% CI)	n(9)	aOR (95% CI)	n(2)	aOR (95% CI)	n(9)	aOR (95% CI)
SMI^a^									
Low	52	5	0.59	3	1.35	0	0	4	1.71
			(0.34–1.74)		(0.43–3.86)		(0-Inf)		(0.96–3.93)
High	103	14	1 (Ref)	6	1 (Ref)	2	1 (Ref)	5	1 (Ref)
TPA^b^									
Low	52	8	1.41	2	0.50	0	0	4	1.71
			(0.73–2.76)		(0.29–1.51)		(0-Inf)		(0.96–3.93)
High	103	11	1 (Ref)	7	1 (Ref)	2	1 (Ref)	5	1 (Ref)
V lateralis m vol									
Operated limb^c^									
Low	52	8	1.39	3	0.97	0	0	5	**2.68**
			(0.76–2.63)		(0.32–2.99)		(0-Inf)		**(1.3–4.66)**
High	103	11	1 (Ref)	6	1 (Ref)	2	1 (Ref)	4	1 (Ref)
Unoperated limb^d^									
Low	52	6	1.21	5	**2.97**	1	2.00	5	**2.68**
			(0.56–2.63)		**(1.1–8.03)**		(0.4–3.09)		**(1.3–4.66)**
High	103	13	1 (Ref)	4	1 (Ref)	1	1 (Ref)	4	1 (Ref)
Post. crural m vol									
Operated limb^e^									
Low	52	7	1.17	3	1.09	1	2.00	3	1.01
			(0.56–2.63)		(0.37–3.17)		(0.4–3.09)		(0.23–2.88)
High	103	12	1 (Ref)	6	1 (Ref)	1	1 (Ref)	6	1 (Ref)
Unoperated limb^f^									
Low	52	6	0.91	3	1.05	1	1.89	4	1.01
			(0.38–2.16)		(0.32–3.38)		(0.68–5.26)		(0.23–2.88)
High	103	13	1 (Ref)	6	1 (Ref)	1	1 (Ref)	5	1 (Ref)

^a^Low: T1 [215–<290], high: T2 [290–<648] and T3 [648–978].

^b^Low: T1 [96.3–<308], high: T2 [308–<398] and T3 [398–604].

^c^Low: T1 [20.2–<41.7], high: T2 [42.7–<51.9] and T3 [51.9–77.2].

^d^Low: T1 [19.1–<39.9], high: T2 [39.9–<49.7] and T3 [49.7–80.2].

^e^Low: T1 [765–<1463], high: T2 [1463–<1922] and T3 [1922–6695].

^f^Low: T1 [753–<1430], high: T2 [1430–<1685] and T3 [1685–6596].

aOR: adjusted odds ratio (adjusted for age, sex, and body mass index); CI, confidence interval; VTE, venous thromboembolism; TKA, total knee arthroplasty; DVT, deep vein thrombosis; PE, pulmonary embolism; SMI, skeletal muscle index; TPA, total psoas area; V lateralis m vol, vastus lateralis muscle volume; Post. crural m vol, posterior crural muscle volume.

**Table 3:** aORs and 95% CIs of radiologically confirmed and symptomatic VTE in simultaneous bilateral TKA.

		Radiologically confirmed PE		Radiologically confirmed right DVT		Radiologically confirmed left DVT		Symptomatic DVT	
	n (106)	n (16)	aOR (95% CI)	Case (11)	aOR (95% CI)	Case (9)	aOR (95% CI)	Case (11)	aOR (95% CI)
SMI^a^									
Low	35	6	1.28	6	1.29	4	1.73	4	1.23
			(0.53–2.76)		(0.43–3.86)		(0.65–4.6)		(0.49–3.1)
High	71	10	1 (Ref)	5	1 (Ref)	5	1 (Ref)	7	1 (Ref)
TPA^b^									
Low	35	5	0.88	3	0.66	3	0.66	4	1.23
			(0.34–1.84)		(0.23–1.87)		(0.23–1.87)		(0.49–3.1)
High	71	11	1 (Ref)	8	1 (Ref)	6	1(Ref)	7	1 (Ref)
V lateralis m vol									
Rt^c^									
Low	35	6	1.26	5	**1.83**	5	**2.97**	5	**1.86**
			(0.6–2.83)		**(1.1–6.48)**		**(1.1–8.03)**		**(1.01–4.66)**
High	71	10	1 (Ref)	6	1 (Ref)	4	1 (Ref)	6	1 (Ref)
Lt^d^									
Low	35	7	1.66	5	**1.86**	4	1.73	5	1.76
			(0.71–3.88)		**(1.04–6.57)**		(0.4–7.09)		(0.57–5.26)
High	71	9	1 (Ref)	6	1 (Ref)	5	1 (Ref)	6	1 (Ref)
Post. crural m vol									
Rt^e^									
Low	35	3	0.39	2	0.3	3	0.96	4	1.09
			(0.1–1.41)		(0.08–1.21)		(0.31–2.4)		(0.23–4.88)
High	71	13	1 (Ref)	9	1 (Ref)	6	1 (Ref)	7	1 (Ref)
Lt^f^									
Low	35	4	0.61	4	1.15	5	**2.94**	3	0.69
			(0.22–1.65)		(0.32–3.38)		**(1.02–5.26)**		(0.23–2.05)
High	71	12	1 (Ref)	7	1 (Ref)	4	1 (Ref)	8	1 (Ref)

^a^Low: T1 [304–<535], high: T2 [535–<638] and T3 [638–1062].

^b^Low: T1 [144.5–<334], high: T2 [334–<414] and T3 [414–642].

^c^Low: T1 [16.8–<32.9], high: T2 [32.9–<40.5] and T3 [40.5–55.1].

^d^Low: T1 [18.9–<49.3], high: T2 [49.3–<54.1] and T3 [54.1–71.9].

^e^Low: T1 [730–<1329], high: T2 [1329–<1659] and T3 [1659–2849].

^f^Low: T1 [845–<1500], high: T2 [1500–<1869] and T3 [1869–3091].

aOR, adjusted odds ratio (adjusted for age, sex, and body mass index); CI, confidence interval; VTE, venous thromboembolism; TKA, total knee arthroplasty; DVT, deep vein thrombosis; PE, pulmonary embolism; SMI, skeletal muscle index; TPA, total psoas area; V lateralis m vol, vastus lateralis muscle volume; Post. crural m vol, posterior crural muscle volume.

## Discussion

The principal finding of this study was that the relative muscle volume of the vastus lateralis at the thigh level was negatively associated with the prevalence of subclinical and symptomatic DVT. To our knowledge, this is the first study to demonstrate the relationship between low relative muscle mass and radiologically confirmed DVT (through CTVPA).

Several limitations of this study should be acknowledged. First, the cross-sectional study design precludes the determination of a causal relationship between muscle volume and DVT prevalence after TKA. Second, patients enrolled in this study were with same race of South Korea, thereby such a huge sexual difference by regional characteristics [16]. Several reports have suggested that there is a strikingly low prevalence of DVT in Asians [17,18]. However, there is a similar prevalence of DVT in Asian ethnic groups and Western populations revealed by routine bilateral venography performed soon after a major joint surgery [19]. The prevalence of thrombi revealed by CTVPA performed during 5–7 days after the operation may be a representative result without any ethnic limitation. Third, a larger sample size may be needed to clarify the risk factors for proximal DVT. Finally, this study did not investigate the preoperative functional performance or muscle strength.

Nevertheless, this study has several strengths. First, CTVPA was used to diagnose VTE, and CTVPA has a high sensitivity for detecting small thrombosis in the calf veins [20]. Second, this is the first study to suggest the protocol of muscle volume measurement with CTVPA, and its clinical usefulness. Third, radiologic assessment of DVT and PE included subclinical thrombotic lesions. According to the conventional mechanical propagation theory [21], postoperative thrombus begins in the calf and extends to the proximal veins. The detachment of the thrombus may lead to PE. Both symptomatic and asymptomatic DVT in the lower limbs seem to increase the risk of PE [21]. Moreover, asymptomatic DVT seems to increase the risk of postthrombotic venous insufficiency in patients undergoing total joint arthroplasty in the long term [22]. Therefore, if asymptomatic DVT is incidentally detected, we suggest providing treatment depending on its location and clinical symptoms.

Some studies showed that the rates of venographic DVT and proximal DVT detected 7–14 days after a major orthopedic surgery in patients who did not receive thromboprophylaxis were approximately 40–60% and 10–30%, respectively [23]. Our study showed a similar rate of DVT to those of other randomized clinical trials. Early diagnosis of DVT and prompt therapy after arthroplasty are essential to reduce the risk of thromboembolic complications. Additionally, identifying the modifiable predisposing factors is also important. The consensus is that the predisposing factors to DVT in Western patients are advanced age, sedentary lifestyle, previous venous disease, congestive heart failure or heart disease or both, prolonged immobilization, presence of malignant disease, obesity, estrogen therapy, and genetic and dietary factors [7]. Postoperative DVT or PE without underlying medical predisposing factors can occur due to vascular stasis, which results from intraoperative vascular disruptions or physical inactivity, and immobilization.

However, no obvious relationship was previously found between muscle volume as a predisposing factor and clinical manifestations such as thrombus location or DVT prevalence [24]. Sarcopenia has been associated with numerous poor outcomes, such as low survival, high infection rate, long length of hospital stay, treatment toxicity, and physical disability, which indicates that persons with sarcopenia are generally less able to deal with stress or disease [12]. The European consensus definition of sarcopenia described CT and magnetic resonance imaging as gold standard methods for estimating muscle mass in research [25]. A widely used parameter for sarcopenia is the SMI, which is the CSA of all skeletal muscles at the L3 level measured with an axial CT scan, as previously stated [26]. The TPA, another commonly used parameter, which is the CSA of the left and right psoas muscles at the L3 level [27], thus includes fewer muscles than the SMI on the same plane.

In the lower extremity, quadriceps strength is a major determinant of physical function in patients who had undergone TKA [28]. The absolute size of each of the four quadriceps femoris muscles decreases with aging. Vastus lateralis tissue has been used to represent the quadriceps femoris muscle in aging-related research [29]. The predilection sites of distal DVT are the muscular, peroneal, and posterior tibial veins in the posterior compartment. Some reports stated that the veins of calf muscles are common sites of acute DVT with risks of propagation and PE [30]. Therefore, we measured the volume of the vastus lateralis as one of the quadriceps muscles at the thigh level and posterior crural muscles at the calf level [29].

This study revealed that patients with lower muscle volume of the vastus lateralis at the thigh level in the nonoperated limb had significantly higher prevalence of both subclinical and symptomatic distal DVT. In the assessment of VTE prevalence according to muscle volume at each level in patients who underwent simultaneous bilateral TKA, similarly to the findings for unilateral TKA, lower muscle volume of the vastus lateralis at the thigh level increased the risk of symptomatic and radiologically confirmed distal DVT. Meanwhile, it was difficult to confirm whether thrombotic events revealed by CTVPA had a positive overall OR with low muscle volume of the operated limb. These findings might result from measurement variations including tourniquet use, vascular congestion, and disuse muscle atrophy.

We hypothesized that mechanical measures together with a large volume of calf muscle have a direct effect on DVT prophylaxis by improving postoperative vascular flow and venous circulation in calf veins. However, proximal muscle at the thigh level, not at the calf level, correlated significantly with the occurrence of distal DVT.

In conclusion, the relative muscle volume of the vastus lateralis at the thigh level was negatively associated with the prevalence of symptomatic and radiologically detected DVT, supporting the finding that low thigh muscle mass is an independent risk factor for VTE in the postoperative period of TKA.

## Supporting information

S1 Table Representative data of patients’ demographics and measurements. (XLSX)
